# Letter to the Editor Re: Kipperman, B.S. and German, A.J. *Animals* 2018, *8*, 143

**DOI:** 10.3390/ani8100179

**Published:** 2018-10-16

**Authors:** Katharine M. Watson

**Affiliations:** Department of Epidemiology and Biostatistics, Indiana University School of Public Health, Bloomington, IN 47405, USA; kamiwats@iu.edu; Tel.: +1-508-292-0168

**Keywords:** companion animal, obesity, overweight, One Health, human–animal bond, medical record

## Abstract

A recent opinion paper by Kipperman and German (2018) discussed the increasing prevalence of pet obesity, the risk factors contributing to this increase, and the role of veterinarians in helping manage pet obesity. They described the problem as a One Health problem as it has been previously characterized. Kipperman and German also reported a sample of medical records from their referring veterinarians wherein a surprisingly small number of veterinarians recorded information about pets’ body weight or discussions with owners about pet obesity. From their sample, they concluded that general practice veterinarians are not meeting their professional and ethical obligations to recognize and address pet obesity. This letter discusses reasons why veterinarians may not be adequately addressing the pet obesity problem. A similar situation exists in human medicine. Numerous studies in the human field have revealed some of the reasons many physicians do not address obesity with their patients. As it is likely that veterinarians have similar reasons for avoiding the obesity issue, obstacles identified by physicians in encountering overweight obesity are reviewed in this letter.

## Dear Editor,

I read, with interest, Kipperman and German’s opinion paper [1] describing the problem of pet overweight and obesity. This letter expands on the authors’ assertion that small-animal veterinarians are not identifying and discussing pet overweight and obesity with clients. The authors base this conclusion on records from referring veterinarians for 148 dogs over a 12-month period. In this sample, 70% of the records included documentation of body weight, 29% included a qualitative assessment of body condition, and only one record included the patient’s body condition score (BCS) [1]. Based on 11 years’ experience as a veterinarian working in referral and general practices, I was surprised at the low number of veterinarians reporting BCS and wonder how representative Kipperman and German’s sample is of the small-animal general practice veterinarian population. 

Most veterinary electronic medical record software now used by veterinarians in the developed world include an entry field for pets’ BCS. In most cases, veterinarians are prompted to enter data in this field. If Kipperman and German’s finding that most veterinarians are not frequently recording BCS or body weight extends to the larger veterinarian population, there may be causes other than poor recognition of pet overweight and obesity.

First, it can be difficult to speak with owners about overweight and obesity as some owners react negatively toward these comments. Veterinarians may recognize the problem and refrain from discussions about pets’ body weight. Second, owners may access their pets’ medical records and I have observed that veterinarians tend to avoid entering comments that may be interpreted negatively by owners. For example, I recall one of my first employers, when I was a new graduate, reprimanding me for recording a pet’s body condition score as eight out of nine. He explained that the owner could read the record and would be insulted.

A similar situation of underreporting of obesity by human physicians has been described by many authors. Physicians are expected to discuss obesity with affected patients and, in the United States, are mandated to document when a patient’s body mass index (BMI) is classified as obese. However, numerous studies have found, like Kipperman et al., that human physicians are under-reporting findings of overweight and obesity. For example, Kraschnewski et al. (2013) found that prevalence of human overweight or obesity increased from 52.1% to 63.3% between the periods 1995–1996 and 2007–2008 in the United States. However, only 39.9% of obese patients presenting in 1995–1996 and 29.9% presenting in 2007–2008 received weight counseling [2]. 

A qualitative study of Australian physicians examined barriers to discussions about weight between patients and physicians. Common barriers cited by the study included: uncertainty about appropriate language, lack of time, concerns about compromising patient trust or rapport, concerns about patients’ readiness to discuss their weight, concerns about how patients’ mental health may be impacted by discussing a potentially difficult subject, and lack of effective treatment or referral options [3]. A cross-sectional study of 500 United States physicians (with a 53% response rate) aimed to assess physicians’ attitudes regarding morbidly obese patients [4]. Many physicians surveyed described the following negative attitudes and barriers to discussions about obesity: patients lack discipline to lose weight (78%), patients want an easy solution (71%), dealing with obesity and weight loss is frustrating (66%), patients do not have time to exercise (62%), patients have poor eating habits (54%), patients’ weight prevents them from exercising, patients lack motivation to lose weight (54%), obesity treatment is often ineffective (51%), and reimbursement for weight loss discussion is inadequate (45%) [4].

Numerous studies have demonstrated that patients desire weight management guidance. Ragsdale et al. (2017) evaluated perspectives of Hispanic patients in the United States and found that patients want their doctors to give more help and advice about managing obesity [5]. Many said their doctors had not raised the subject of weight management and that they would value assistance with specific weight loss goals, dietician referrals, education, and encouragement [5]. 

Further studies, perhaps similar to the studies undertaken on physicians’ attitudes toward obesity discussion and management, would help (1) describe the extent to which veterinarians are not recording or discussing overweight and obesity with clients, (2) determine potential influences on veterinarians’ attitudes toward patient weight management, and (3) find effective means to help veterinarians more effectively report and discuss pets’ body weight. The multidisciplinary One Health approach, which can bridge gaps between human and animal medicine, may be an ideal way to provide new insights and solutions to both the human and animal obesity problems. 

## Abbreviation

The following abbreviations were used in this manuscript:

BCS	body condition score
BMI	body mass index
